# Telomerase activity, apoptosis and cell cycle progression in ataxia telangiectasia lymphocytes expressing TCL1

**DOI:** 10.1038/sj.bjc.6601213

**Published:** 2003-09-09

**Authors:** C Gabellini, A Antonelli, P Petrinelli, A Biroccio, L Marcucci, G Nigro, G Russo, G Zupi, R Elli

**Affiliations:** 1 Experimental Chemotherapy Laboratory, Regina Elena Cancer Institute, Rome, Italy; 2 Cellular Biotechnology and Hematology Department, University ‘La Sapienza’, Rome, Italy; 3 Pediatric Institute, University ‘La Sapienza’, Rome, Italy; 4 Istituto Dermopatico dell' Immacolata, Rome, Italy

**Keywords:** ataxia telangiectasia, TCL1, telomere, apoptosis, cell cycle

## Abstract

Individuals affected by ataxia telangiectasia (AT) have a marked susceptibility to cancer. Ataxia telangiectasia cells, in addition to defects in cell cycle checkpoints, show dysfunction of apoptosis and of telomeres, which are both thought to have a role in the progression of malignancy. In 1–5% of patients with AT, clonal expansion of T lymphocytes carrying t(14;14) chromosomal translocation, deregulating TCL1 gene(s), has been described. While it is known that these cells can progress with time to a frank leukaemia, the molecular pathway leading to tumorigenesis has not yet been fully investigated. In this study, we compared AT clonal cells, representing 88% of the entire T lymphocytes (AT94-1) and expressing *TCL1* oncogene (ATM^−^ TCL1^+^), cell cycle progression to T lymphocytes of AT patients without *TCL1* expression (ATM^−^ TCL1^−^) by analysing their spontaneous apoptosis rate, spontaneous telomerase activity and telomere instability. We show that in ATM^−^ TCL1^+^ lymphocytes, apoptosis rate and cell cycle progression are restored back to a rate comparable with that observed in normal lymphocytes while telomere dysfunction is maintained.

Ataxia telangiectasia (AT) is an autosomal recessive disease characterised by progressive cerebellar degeneration, variable immunodeficiency, genomic instability and susceptibility to cancer, especially lymphoid malignancies. The product of *ATM* gene, which is mutated in individuals affected by AT, belongs to a well-conserved family of protein kinases. It is involved in the control of the cell cycle, in the processing of DNA damage and in the maintenance of genome integrity (caretaker gene) (Shiloh and Kastan, 2001). In particular, the Atm protein is homologous to Tel1p, which is essential for telomere maintenance in *Saccharomyces cerevisiae* (Greenwell *et al*, 1995; Pandita, 2002), and may regulate the structure and the function of telomeres through a not yet fully understood mechanism; indeed, AT cells have an accelerated rate of telomere loss and show chromosomal end-to-end telomeric fusions/associations (*tas*), in spite of having normal telomerase activity (Pandita *et al*, 1995; Metcalfe *et al*, 1996). Furthermore, Atm might play a role in apoptosis as suggested by its involvement as a substrate of caspase 3 (Smith *et al*, 1999); however, different cell types that lack the *ATM* function show apoptotic behaviour not always consistent (Meyn, 1999; Bebb *et al*, 2001).

Tumours of the lymphoid system are very frequent in AT patients. Among them T-prolymphocytic leukaemia (T-PLL), a very rare lymphoid neoplasia in general population, is also quite often observed in AT patients (Taylor *et al*, 1996). These T-PLLs in ATs have been well characterised in the past by several groups including us, and are quite invariably preceded by a T cell clonal expansion identifiable cytogenetically to carry a t(14;14)(q11;q32.1) translocation or an inv(14)(q11;q32.1) inversion or a t(X;14)(q28;q32.1) translocation. With the time, usually years, these clonal cells are able to expand until they represent up to 90% of the circulating T lymphocytes and ultimately to evolve into an overt leukaemia. These chromosomal rearrangements, at molecular level, bring regulatory elements of the TCR*α* (located on14q11) to a new position on 14q32.1 or Xq28 resulting in activation of usually silent genes in normal mature T cells. These genes belong to the TCL1 gene family: *TCL1* and *TCL1b* are located on 14q32.1 and *MTCP1* is on Xq28 (Stern *et al*, 1993; Virgilio *et al*, 1994; Pekarsky *et al*, 1999). Tcl1, Tcl1b and Mtcp1 identify a new family of proteins since they are practically identical, either by sequence or structure, and are usually absent in mature normal circulating T cells. The consequences of *TCL1* expression are due to its binding to the serine/threonine kinase Akt (also called protein kinase B, PKB), by increasing kinase activity and enhancing Akt nuclear translocation (Laine *et al*, 2000; Pekarsky *et al*, 2000). Furthermore, TCL1 family gene members have oncogenic ability, as clearly demonstrated by four different transgenic animal models in which overexpression of these genes, in either T or B cells, causes T-PLL, B-chronic lymphocytic leukaemia (B-CLL) or mature B-cell lymphoma (Gritti *et al*, 1998; Virgilio *et al*, 1998; Bichi *et al*, 2002; Hoyer *et al*, 2002).

We previously described a large nonleukaemic clone carrying the chromosomal tandem translocation t(14;14)(q11;q32) in an AT patient (AT94-1), who, at present, has not yet shown any sign of malignancy. The clonal cells are characterised by the overexpression of the *TCL1* oncogene (Narducci *et al*, 1995), a high rate of spontaneous chromosome instability (especially of *tas*) (Petrinelli *et al*, 2001) and hypersensitivity to the topoisomerase II inhibitor VP16 (Petrinelli *et al*, 1996). These clonal cells represent a very useful tool to study the biological consequences of *TCL1* overexpression in the context of ATM deficiency since they are not hampered by ectopic transfection. In this study, we report that in this AT cell clone, even when VP16-damaged, the apoptosis rate and the cell cycle progression are restored back to a rate comparable with that observed in normal lymphocytes while telomere dysfunction is maintained.

## MATERIALS AND METHODS

### Lymphocyte cultures

Peripheral blood samples were obtained from the AT94-1 patient, from six normal controls and from two AT controls (AT95-1 and AT95-2). AT94-1 and AT95-2 were reported as AT22RM and AT28RM, respectively, by Gilad *et al* (1996).

To evaluate the spontaneous rate of *tas*, chromosome preparations were performed by standard method, and analysed on G-banded metaphases by two independent observers.

To evaluate telomerase activity, telomere length, cell cycle progression and apoptosis, blood was defibrinated and mononuclear cells isolated by centrifugation on a layer of histopaque 1077 (Sigma Diagnostic, Milan, Italy). Purified lymphocytes (1 × 10^6^ ml^−1^) were incubated for 72 h in RPMI 1640 (Sigma) supplemented with 10% fetal calf serum, penicillin, streptomycin and PHA (5 *μ*g ml^−1^). To evaluate the effects of the topoisomeraseII inhibitor VP16 on cell cycle and apoptosis, VP16 (final concentration 0.1 *μ*g ml^−1^) was added to purified lymphocyte cultures 48 h before harvesting (Petrinelli *et al*, 1996).

### Telomerase activity and telomere length

Telomerase activity was measured with the PCR-based TRAP (telomeric repeat amplification protocol) kit (Intergen Company, Oxford, UK) according to the manufacturer's instructions. To define the sensitivity of the method and the semiquantitative relationship between protein concentration and ladder band intensity, different amounts of protein extract (from 0.1 to 2 *μ*g) were used for each purified lymphocyte culture and for all assays. The appropriate protein concentration to produce a linear response was 1 *μ*g of the total protein. In all cases, reaction products were amplified in the presence of a 36 bp internal TRAP assay standard (ITAS), and each extract was tested for RNAse sensitivity, incubating samples with 20 ng RNAse (Sigma) for 30 min at 37°C before extension/amplification reaction. Telomeric repeat amplification protocol assay included a sample without cell lysate as negative control and a positive control sample provided by the kit. Telomere length was evaluated by Southern blot as previously reported (Biroccio *et al*, 2002). Briefly, total DNA was isolated using standard procedure. For each sample, 15 *μ*g of DNA were digested with 40 U of *Hinf*1 and electrophoresed on 0.8% agarose gel. DNA was denatured, neutralised, transferred to a nylon membrane (Hybond N, Amersham International, Buckinghamshire, UK) and cross-linked with ultraviolet light. The membrane was hybridized with 5′-end [*γ*-^32^P] deoxyadenosine triphosphate-labelled telomeric oligonucleotide probe (TTAGGG)_3_ at 42°C for 4 h in a rapid hybridization buffer (QuikHyb Hybridization Solution, Stratagene, La Jolla, USA). After washing, the filters were autoradiographed (Hyperfilm-MP; Amersham) with an intensifying screen at −80°C for 24 h. The autoradiographs were scanned and the mean telomere length was calculated as reported by Harley *et al* (1990).

### Cell cycle analysis and detection of apoptosis

The percentage of cells in the different phases of cell cycle was determined by evaluating DNA content. Treated and untreated cells (2 × 10^6^) were collected at 72 h of culture, washed with PBS and fixed with 2 ml of 70% ethanol. After washing twice in PBS, the cells were stained with a solution containing 50 *μ*g ml^−1^ propidium iodide (PI) and 75 kU/ml RNAse in PBS for 30 min at room temperature. A total of 20 000 events per sample were acquired by FACSCalibur (Becton-Dickinson) using a doublet discrimination module (DDM). The percentages of cell cycle distribution and subG1 apoptotic cells were calculated on linear PI histograms using the mathematical software ModFit. Apoptotic cells were also detected by using the APO-BRDU™ kit (BioSource International, Camarillo, California), a terminal deoxynucleotide transferase (TdT)-mediated Br-dUTP nick-end labelling (TUNEL), according to the manufacturer's instruction. Briefly, 2 × 10^6^ of treated and untreated cells were collected at 72 h of culture, washed with PBS and fixed with 2 ml of 70% ethanol. The fragmented DNA was revealed incubating the samples with the labelling reaction mix for 1 h at 37°C; the BrdU-labelled cells were then stained with a fluorescein-labelled anti-BrdU antibody solution for 30 min at room temperature, in the dark. To determine DNA content, a PI/RNAse solution was added to FITC-labelled cells and incubated for 30 min at room temperature, in the dark.

### Statistical analysis

Statistical evaluation of apoptosis and cell cycle data was performed by Student's *t*-test.

## RESULTS

### Telomerase activity and telomere length in AT lymphocytes expressing TCL1

In order to study the correlation between chromosome end-to-end fusions (*tas*) and telomere metabolism, we evaluated telomerase activity and telomere length in PHA-stimulated lymphocytes characterised by different *tas* frequency. The cells used were: AT lymphocytes with *TCL1* expression (AT94-1), which are characterised by a high frequency of spontaneous *tas* (Table 1

**Table 1:** Spontaneous telomeric associations (*tas*) in metaphases with t(14;14) from AT94-1 lymphocytes

**Date**	**No. cells analysed**	**Metaphases with *tas***	**Metaphases with subclonal rearrangements**
2001	52	tas(21;22)(p11;p11)	−22,+mar1[2]
		tas(18;21)(p11;p11)	i (21)(q10)[5]
		del(7)(p11) tas(18;13)(q23;q34)	i (21)(q10),tas(10;15)(p15;p11)
		tas(10;21)(p15;p11)	i (21)(q10),tas(1;18)(p36;p11)
		tas(5;9)(p15;q34)	+6, i (21)(q10)
		tas(5;22)(p15;p11)	
		+5,−22, tas(11;21)(q25;q22)	
		−18, i(18)(q10), tas (dert[14;14][q11;q32];7)(p11;q36)	
		t(5;13)(q12;q12),	
		tas(13;17)(p11;p13),tas(9;18)(q34;p11)	

2002	56	tas(4;21)(p16;p11)	−22,+mar1 [2]
		tas(5;6)(p15;p25)	i (21)(q10) [3]
		tas(21;21)(p11;q22)	i (21)(q10),inv(3)(p21q11)
		tas(4;13)(p16;q34)	
		+X, tas(12;18)(q24;p11)	
		tas(21;22)(p11;q13)	
		tas(2;18)(q37;p11)	
		tas(5;15)(p15;p11)	
		tas(4;21)(p16;q22)	

**Figure 1:**
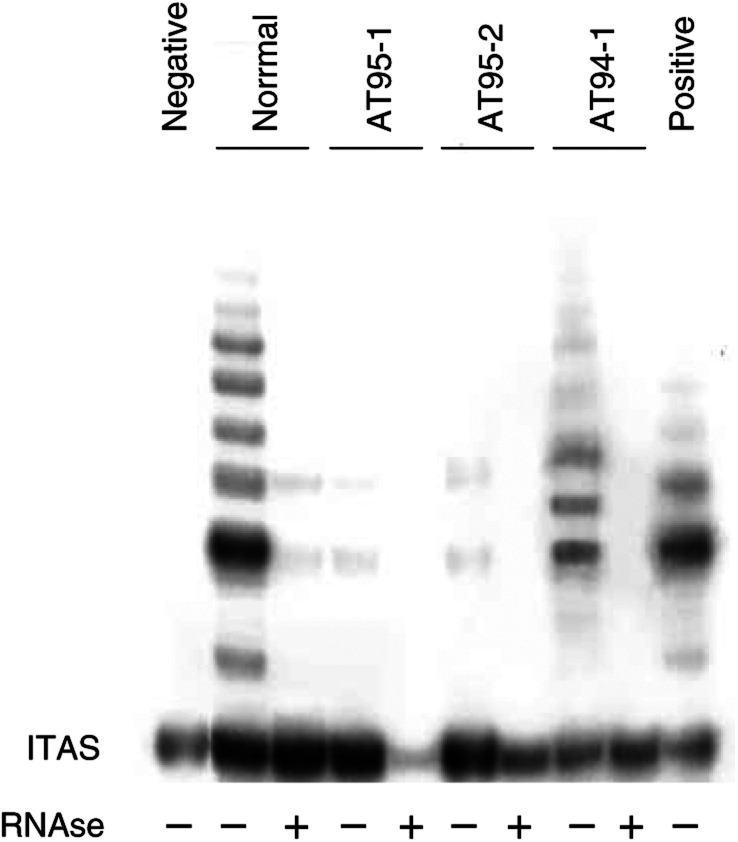
TRAP assay to evaluate the effect of ATM status and *TCL1* expression on telomerase activity in human PHA-stimulated lymphocytes. From left to right: a negative control; a sample of normal lymphocytes; two samples of AT lymphocytes (AT95-1, AT95-2); a sample of AT lymphocytes expressing *TCL1* (AT94-1); a positive control.

The metaphases with the t(14;14) rearrangement represented 80 and 73% of total AT94-1 lymphocytes, respectively, in 2001 and in 2002. Metaphases with sporadic nonclonal rearrangements were also observed (data not shown). The cytogenetic follow-up of AT94-1 from 1995 to 2000 has been previously reported (Petrinelli *et al*, 2001).

); Ataxia telangiectasia lymphocytes without *TCL1* expression (AT95-1 and AT95-2) showing sporadic *tas*; and normal lymphocytes, with no detectable *tas*. The telomerase activity was evaluated by a semiquantitative TRAP assay. Figure 1

shows that the loss of the ATM function alone determines a marked decrease in telomerase activity (see AT95-1 and AT95-2), while the expression of *TCL1*, characteristic of AT94-1 clonal lymphocytes, is able to restore telomerase activity up to the level observed in normal lymphocytes. The internal telomerase assay standard (ITAS) was amplified to the same extent in all cell cultures, excluding the presence of *Taq* polymerase inhibitors in AT lymphocytes without *TCL1* expression.

We then tried to determine whether the increase of telomerase activity observed in AT cells expressing *TCL1* was associated with reduced telomere shortening. Terminal restriction fragments (TRFs) were obtained by Southern blotting in the same cells analysed by TRAP assay (Table 2

**Table 2:** Terminal restriction fragments calculated by Southern blot analysis of normal, AT and TCL1 espressing AT lymphocytes hybridized with the telomeric repeats (TTAGG)_3_

**Cells**	**Telomere length (kbp)**	
	**Min**	**Max**
AT^+^ TCL1^−^	12.2±0.2	19.0±0.2
AT^−^ TCL1^−^	10.6±0.2	15.6±0.1
AT^−^ TCL1^+^	10.5±0.2	15.7±0.1

The results are the mean values of three experiments.

). TRFs in AT lymphocytes without *TCL1* expression were shorter (mean value 13.1 kb) than in normal lymphocytes (mean value 15.6 kb). AT94-1 lymphocytes (expressing *TCL1*) showed a telomere length range superimposable to that observed in AT lymphocytes not expressing *TCL1*, although these two kinds of AT cells have different telomerase activity and different *tas* frequency.

### Decreased apoptosis and restoration of cell cycle progression in AT lymphocytes expressing TCL1

**Figure 2:**
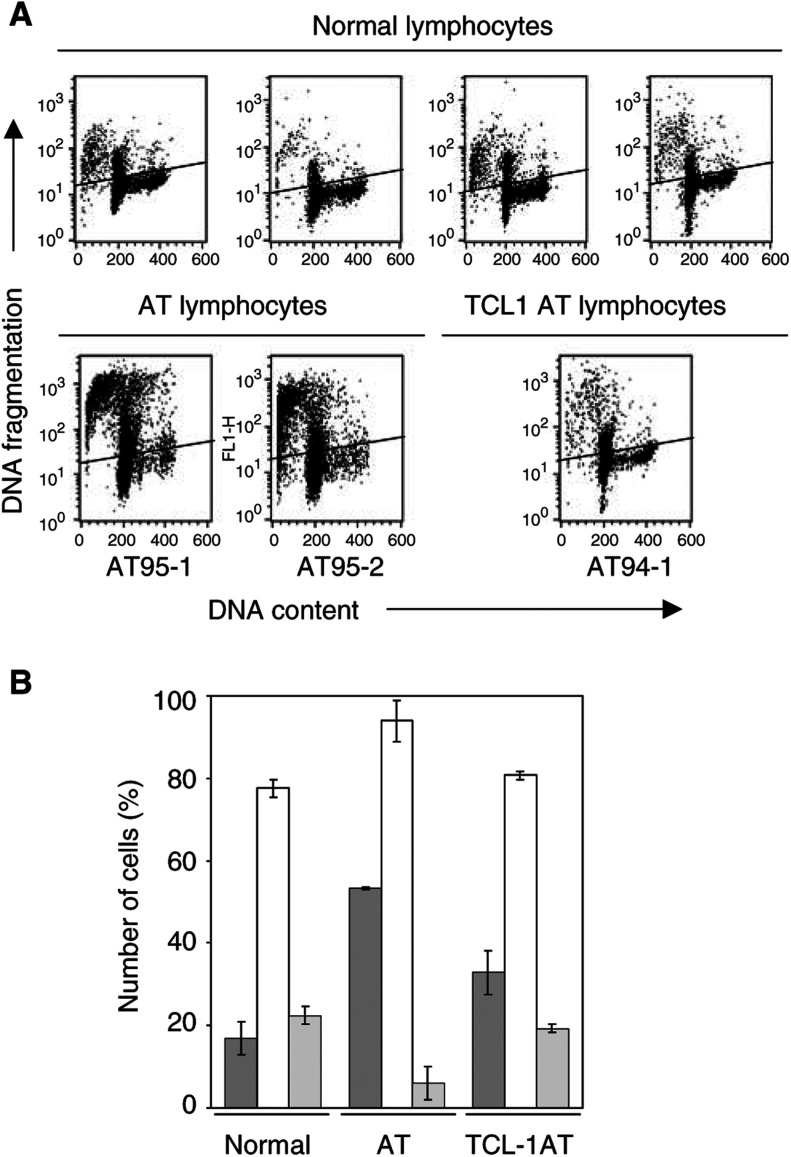
Evaluation of apoptosis and DNA content of different types of lymphocytes. (**A**) Results of a representative TUNEL assay performed on four samples of normal lymphocytes, two of AT lymphocytes (AT95-1 and AT95-2) and one of *TCL1* expressing AT lymphocytes (AT94-1). (**B**) TUNEL-positive cell percentage (dark grey columns), cell percentage in the G_0_/G_1_ phase (white columns) and in the S–G_2_/M phase (grey columns) of cell cycle. Each experiment was performed at least three times.

In order to ascertain whether the differences in telomere metabolism in cells that have lost the *ATM* function are due to different rates of proliferation and/or apoptosis, we analysed these two features comparing AT lymphocytes with and without *TCL1* expression. A flow cytometric TUNEL assay, performed in a biparametric analysis with DNA content (Figure 2A

), was carried out in four different samples of normal lymphocytes, in two AT lymphocytes (AT95-1, AT95-2) and in AT94-1 lymphocytes. Each sample was analysed at least three times. Figure 2B shows the mean values of apoptotic cells (dark grey columns) together with the mean percentages of cells in the different phases of cell cycle (G_0_/G_1_, white columns; S-G_2_/M, grey columns). Ataxia telangiectasia lymphocytes without *TCL1* expression (see AT) were characterized by a significant (*P*<0.01) spontaneous activation of the apoptotic programme (53.3±0.4) if compared with normal lymphocytes (17.0±2.1) and showed a strong proliferative block in the G_0_/G_1_ phase. The expression of *TCL1* in AT cells determined a decrease of the apoptotic rate (32.9±5.3) and was able to restore a ‘normal like’ cell cycle by driving the AT cells in the S phase. In fact, the differences in the percentage of cells in G_0_/G_1_ and S–G_2_/M phases between normal and AT94-1 lymphocytes were not significant (*P*>0.05).

We next evaluated the same parameters in the same cells after DNA damage induction by VP16 treatment (Table 3

**Table 3:** Effect of *TCL1* expression on apoptosis and on cell cycle in AT lymphocytes after treatment with VP16

**Cells**	**S–G_2_/M accumulation**	**Apoptosis increase**
AT^+^ TCL1^−^	2.01±0.14	1.65±0.08
AT^−^ TCL1^−^	1.24±0.13	0.90±0.06
AT^−^ TCL1^+^	2.33±0.2	1.81±0.39

Data are presented as a ratio between the percentages of treated *vs* untreated cells, respectively, in S–G_2_/M compartment and in apoptosis. Values are the mean and s.e. of five experiments for AT^+^ TCL1^−^ (normal control), five experiments for AT^−^ TCL1^−^ (AT control) and three experiments for AT^−^ TCL1^+^ (AT94-1).

). As expected, in normal lymphocytes (AT^+^ TCL1^−^) VP16 treatment induced a significant (*P*<0.01) increase of the percentage of cells in S+G_2_/M compartement and in apoptosis, while we observed no significant (*P*>0.05) change for both parameters in control AT lymphocytes (AT^−^ TCL1^−^). On the contrary, in AT lymphocytes expressing *TCL1* (AT^−^ TCL1^+^) VP16 treatment determined an increase of apoptosis rate and of percentage of cells in the S–G_2_/M compartment comparable to those observed in normal lymphocytes.

## DISCUSSION

In this study, we demonstrated that AT94-1 T lymphocytes have lost the general growth disadvantage caused by the lack of the *ATM* function. The dramatic reduction of cells in the S and G_2_ phases of the cell cycle, characteristic of nonclonal AT lymphocytes, was not observed in AT94-1 lymphocytes since these cells showed a cell cycle distribution similar to that observed in normal cells. Furthermore, our results suggest that *TCL1* expression is correlated with a reduced apoptotic rate in the apoptotic-prone ATM^−^ cells (Duchaud *et al*, 1996). The differences in cell proliferation and survival were also evident in cells treated with VP16, a topoisomerase II inhibitor that acts at premitotic phase and induces apoptosis in mitogen-activated T lymphocytes (Sinkule, 1984; Ferraro *et al*, 2000). Indeed, when damaged with VP16, only ATM^−^ TCL1^+^ lymphocytes were able to show an increase, both in apoptosis rate and in percentage of cells in the S–G_2_/M compartment, similar to that observed in VP16-treated normal lymphocytes (ATM^+^ TCL1^−^).

We also showed that AT94-1 cells exhibited telomere shortening, increased telomerase activity and chromosomal instability, especially telomeric end-to-end fusions (*tas*). The coexistence of telomere instability and telomerase activation in AT94-1 cells can be explained by taking into account that telomerase activity does not necessarily result in telomere elongation, in agreement with previous studies (Metcalfe *et al*, 1996). Indeed, telomerase might have a dual function for telomeres, protection and elongation, and could therefore promote cell survival and growth, independently of net telomere elongation (Zhu *et al*, 1999; Blasco, 2002). We also observed that endogenous expression of telomerase in AT94-1 cells is unable to correct the cytogenetic phenotype of telomere instability (i.e. *tas*). In agreement with this, Wood *et al* (2001) showed that the ectopical expression of the human telomerase gene *hTERT* in AT fibroblasts does not correct the telomere end-association defect, despite maintaining/extending telomere length. Thus, *tas* frequency could be dependent on ATM loss, but is telomerase independent. In fact, as suggested by Pandita and Dhar (2000), ATM could be involved in telomere-mediated function through telomere–nuclear matrix interaction. Therefore, AT94-1 lymphocytes regain telomerase activity despite high frequency of *tas*, show low spontaneous apoptosis rate and restore cell cycle progression. It is then *TCL1* expression that confers these advantages to ATM^−^ cells. Even though we cannot exclude that other undetected genetic alterations might be present in the clonal cells, *TCL1* seems to play an important role in these features. This idea is consistent with the observation that *TCL1* is uniquely expressed in AT94-1 lymphocytes and not in other AT or normal T lymphocytes (Narducci *et al*, 1995,^2000^) and that it has a proliferative and antiapoptotic role as demonstrated by recent biochemical findings and studies in animal models. More specifically, biochemical evidence demonstrates that Tcl1 acts *in vivo* as a coactivator of the Akt protein, enhancing its kinase activity and mediating its nuclear translocation (Pekarsky *et al*, 2000; Kunstle *et al*, 2002). Akt, in turn, has a central role in the regulation of several signalling pathways controlling cell survival and proliferation in T cells. Indeed, activated Akt enhances human telomerase activity through phosphorylation of the hTERT subunit (Kang *et al*, 1999), inhibits the proapoptotic factor Bad (Nicholson and Anderson, 2002) and, in T lymphocytes, has a key role in G_1_–S phase progression through the regulation of pRB (retinoblastoma protein) phosphorylation (Brennan *et al*, 1997). The same pathway Akt/pRB/E2F is required also to protect activated peripheral T cells from bcl-2-independent apoptosis (Lauder *et al*, 2001). The overexpression of this gene in T or B lymphocytes of transgenic mice induces, respectively, a T-PLL, B-CLL or mature B-cell lymphoma (Virgilio *et al*, 1998; Bichi *et al*, 2002; Hoyer *et al*, 2002). Interestingly, Fears *et al* (2002) reported that one cell line, derived from a relapse in a child with acute lymphatic leukaemia (ALL), showed TCL1 overexpression and a much higher rate of proliferation when compared to a previously established cell line from the same patient. The increase in TCL1 expression correlates with disease progression and more aggressive phenotype in this child.

In conclusion, clonal T cells carrying t(14;14) chromosomal translocation and expressing *TCL1* seem to be able to restore some of the biological properties that are lost in AT cells; this might explain why these T cells have greater expansion and survival rate and are most often observed in AT patients. This assumes even more relevance if we consider that a similar situation of ATM biallelic loss and TCL1 overexpression, other than in AT patients, exists also in several sporadic human cancer types, such as T-PLL, B-CLL and mantle cell lymphoma, suggesting that these two genes cooperate in the development of certain lymphoid tumours.
